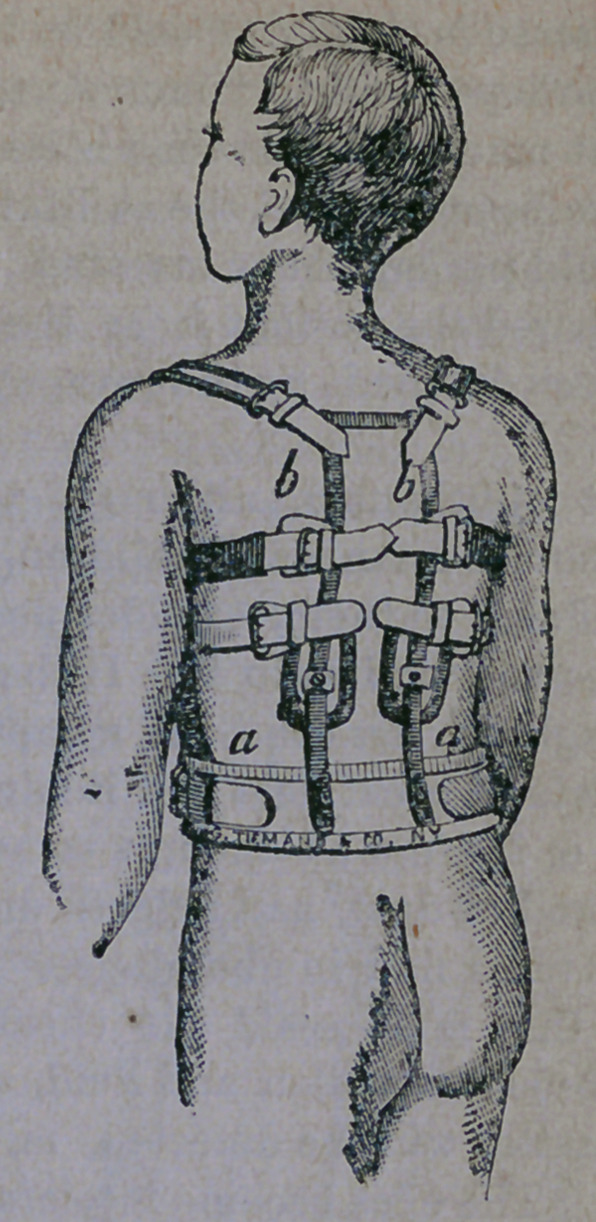# Spinal Curvature

**Published:** 1873-10

**Authors:** 


### SPINAL CURVATURE.

The spinal column is composed of twenty-four hollow bones, named *vertebræ*, and joined together by means of twenty-three slightly elastic cushions, called *inter-vertebral cartilages*, the structure giving protection to the spinal cord, and an erect position to the body. The adult spine has three curves,—two slight ones in front, and a large one behind. These curves seem to be intended to afford space and support to the vital organs within the chest and abdomen, while they expose the column itself to injury, and to the necessity of requiring numerous and powerful muscles to keep it in an erect position. These muscles, when contracting irregularly, or when acting upon softened, diseased or otherwise weakened vertebræ or cartilages, produce unnatural and unsightly curvatures, the two principal forms of which we are about to describe in this article.

*Lateral Curvature*, is that form of spinal distortion, in which the bend is toward either side, and is produced by a variety of causes, the most common of which are falls or blows upon the spinal column ; affections of the muscles, as paralysis, spasmodic contraction, etc. ; debility, from protracted sickness, or inherited feeble or scrofulous constitution ; softening of the bones or cartilages, as in the disease known as "rickets," etc. Perhaps the most frequent cause is a persistence of the child in maintaining, for hours at a time, the erect position, after the muscles of the back have become tired, keeping them continually upon the stretch, until they become so exhausted as to be partially or completely incapable of affording sufficient support to the column, when it naturally gravitates to the right or left. Or, should the muscles upon one side become enfeebled, as is very often the case in delicate children, then the spine is drawn in the direction of the antagonizing ones, upon the opposite side, so producing a curvature in that direction. This difficulty is often met with in anæmic children, who are required, by thoughtless parents, to sit at a desk in the school-room, hour after hour, with inadequate support for their backs, when they *should* be in the open air, free to run and play, and so gain an appetite for the wholesome food necessary to place the requisite strength in their muscles, to keep their spines in the position intended by nature. In *rachitis*, a disease in which the bones of the child become soft and yielding, from want of sufficient earthy deposit in their composition, the vertebræ become so weak as to be unable to resist the amount of pressure exerted upon them by the muscles of the back, when the spine becomes drawn, not only sidewise, but backwards, also, thus giving rise to the very worst form of lateral curvature with which we have to contend. Of course, the extent of a curvature depends measurably upon the health of the child, and the good sense of its parents. If the deviation is discovered early, and the child placed *immediately* under the care of a competent surgeon, it is usually at once arrested; otherwise, it may occupy one-half, two-thirds, or even the entire length of the spinal column. Generally, it is first discovered on the right side, between, or just below, the shoulder blades, when the *deltoid, spinate, trapezius,* and *rhomboid* muscles, overpowering their fellows upon the oppoite sides, drag the vertebræ and everything connected with them, over to the other side, producing a condition of affairs seen in the cut below:

The spine is always shortened in proportion to the amount of curvature present, the chest is altered in appearance, while the shoulder drops to complete the unseemly deformity. In the earlier stages of the affection, the curvature is produced by the absorption of the cartilage between the vertebræ, upon the side toward which the column leans; but, as it advances, the bones themselves become involved in the difficulty, rendering the deformity still greater and the means of cure more difficult and irksome.

The symptoms of approaching curvature of the spine are recognized in the child complaining of fatigue in walking; pain in the back after sitting in a chair a while, easily fatigued at play, occasional suffering from palpitation of the heart, constipation of bowels, and the countenance exhibiting a pale and careworn appearance. As the disease advances, they will complain of tenderness of the spine, when touched with the fingers, or even when coming in contact with the bed, and soon, the slight deviation of the spinal processes, to the right or left, will be observed, if the back is examined for the purpose.

It occurs most frequently in young girls, from five to twenty years of age, and especially in those that possess naturally feeble constitutions.

In almost all instances, the cure of lateral curvature of the spine is easy of accomplishment, if recent, and the patient in moderately good health. Should the difficulty depend upon some latent disease, or a naturally enfeebled or scrofulous constitution, means must be sought for invigorating the general health, before we can expect much benefit to be derived from mechanical treatment. The patient should therefore be placed under the care of a competent physician, who will, when the improved health of the patient permits, adjust a suitable brace, the object of which shall be to remove the weight of the head and shoulders from the weakened spine, and place it upon the hips, by means of a crutch-like, steel brace, running from a well-padded, steel band encircling the hips, up to each arm-pit. These crutches, while they support the weight, also push the fallen shoulder in position, and maintain it there. Then, a strong, elastic, rubber webbing is fastened, by means of suitable buttons, to the brace in such a manner, as to exert a constant and even pressure upon the curved portion of the spine, in a contrary direction to that in which it is leaning. A better idea of such an apparatus however, is given by means of the excellent electrotype shown below:

The apparatus is light, strong, and well padded where ever it comes in contact with the body ; and, although the wearing of it may prove irksome for a few days, yet, such is the comfort derived from its use, the patient soon could not be persuaded to go without it. It is not expected that the same brace would be adapted, in every particular, to every patient suffering from lateral curvature. Not by any means. We only give the general plan of construction of such implements, varying them to suit the requirements of each particular case.

We are confident in our belief, and have demonstrated it over and over again, that nearly, if not quite all, the lateral curvatures of the spine, common to childhood, and in many instances those of adults, are amenable to skillful physical and mechanical treatment. Surgeons can be found in almost all of our larger cities, who have had much experience in the treatment of these affections, and who have special hospitals for the accomodation of just such cases, and by means of the wonderful mechanical appliances which this enlightened age has placed in our hands, we are enabled to remove deformities and restore to health and usefulness, poor, helpless cripples, who aforetime, were miserable in the thought that no means existed for their restoration as useful members of society.

We now come to the consideration of *angular curvature* of the spine, perhaps better known as "Potts' disease." It occurs commonly in children of tender years,—usually from five to ten years of age, those who are poorly fed and clad, or are the offspring of fashionably diseased parents. An attack of cold, a slight local injury to the spine, or the ravages of scrofulous or tubercular disease may spring it into existence. The disease attacks the vertebra itself, which softens and decays, giving rise to that form of disease of the bone known as *caries*. Sometimes the disease is limited to a single bone, and again two or three are involved in the trouble, the inner or thicker portion becoming soft, spongy and finally completely destroyed, when the spine falls forward, above the seat of disease, and bends backward below, forming a right angle. In the most aggravated form, the spine protrudes backward many inches beyond its natural position, forming an unsightly hump upon the back. The ribs are also thrown closer together, causing the breastbone and chest to protrude in front, while the head sinks between the shoulders, to complete the terrible and irremediable deformity.

Pott's disease comes on in such a stealthy manner that it is usually not recognized until it has advanced so far as to render the affection incurable. In the early stages, the child is observed to be in declining health ; is pale, puny, easily fatigued, and has lost all disposition for play. The appetite fails, the gait becomes irregular and tottering, numbness is complained of in the feet, and a sense of heat and discomfort along the spine. The patient is restless at night, has a slight fever, which disappears in the morning, and the urine is scanty. As the disease advances, the pain in the back increases, the debility becomes more marked, the numbness of the extremeties increases, until partial or complete paralysis occurs. If the patient is able to sit up, or is allowed to, the spine is very painful and gradually settles into the deformity before explained.

The treatment is difficult of accomplishment at home, for the reason that the child requires such constant attention and must be kept strictly upon its back, upon a suitable bed, and such appliances used for its comfort and for the correction of the deformity, as could not be afforded save in an institution devoted to the treatment of such cases. The nature of the case requires absolute and complete rest in the recumbent position. And this position *must* be maintained until all trace of the disease is removed, when an apparatus similar to the one here figured is applied and the patient then gradually accustomed to the erect position;

In this arrangement the crutches are supported upon the padded steel band, which rests upon the hips. This removes the weight of head and shoulders from the weak spine. A stout piece of elastic webbing is shaped over the protruding spine, and fastened to the pelvic band by means of steel supports, and thence to the crutch, over the shoulder, by aid of the straps, seen in the engraving. This arrangement exerts a constant pressure upon the protuberance, tending to force it back to its proper position, while the crutches raise the body in front and tend to straighten the deformity in the chest.

As this arrangement would be uncomfortable to sleep in, it is removed after the patient has been duly laid upon his back, in bed, and the stout elastic band shown below, placed over the seat of deformity. This not only gives support to the trunk, but also assists, by its gentle pressure, in restoring the spine to its normal position when all the muscles are relaxed, while the patient is enjoying his sleep.

In the morning the spinal brace is again applied *before* the patient is allowed even to sit up in bed, as great care must be observed not to allow the least weight to fall upon the diseased and weakened bones, else would the inflammation be increased, and the cure greatly retarded.

As the patient grows stronger and straighter, under a healthful diet, suitable tonics, and light gymnastic exercises, a lighter apparatus is then applied, as represented below: when he is allowed to run about without much further attention, only requiring him to wear the apparatus for a year or more, or until all trace of tenderness in the region of the spine has subsided.

Our object in writing and illustrating this article in the manner we have, is to clearly exhibit to parents what these deformities are, and to indicate to them the means of cure. Doubtless, thousands of children are suffering from spinal curvatures whose parents are not conscious of there being anywhere in existence, any means by which their little ones might be restored to health and usefulness. If our article has succeeded in convincing them that cures *can* be accomplished, in the majority of instances, and has clearly indicated to them the manner in which it is done, and through its means parents will be encouraged to seek such relief for their helpless children, our object will be entirely accomplished.

## Figures and Tables

**Figure f1:**
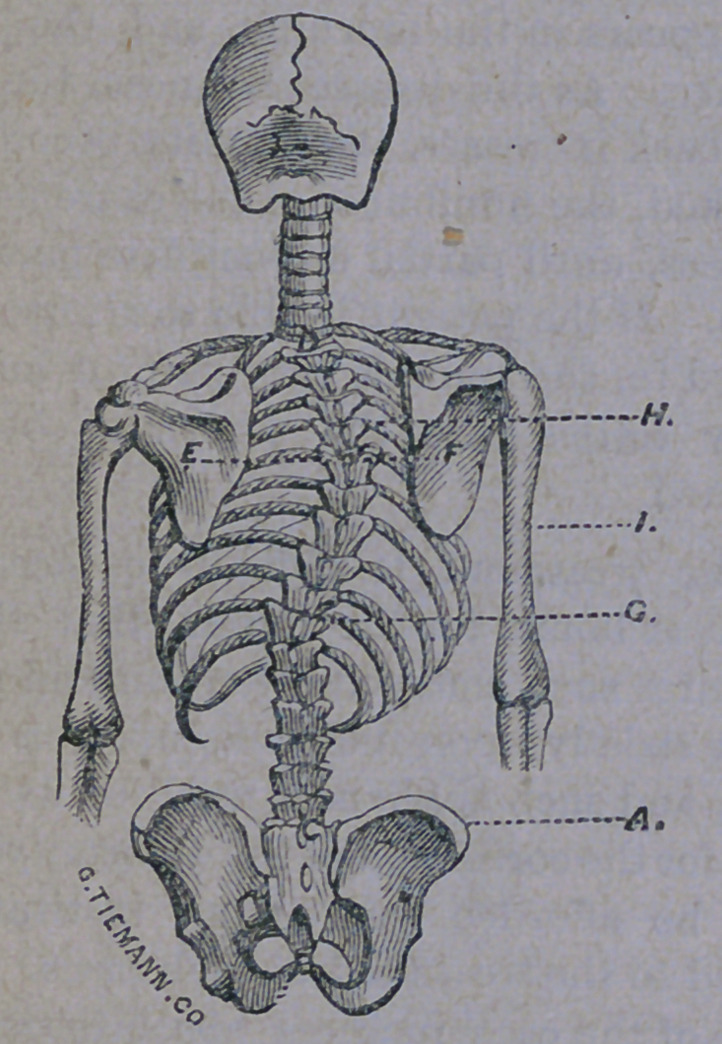


**Figure f2:**
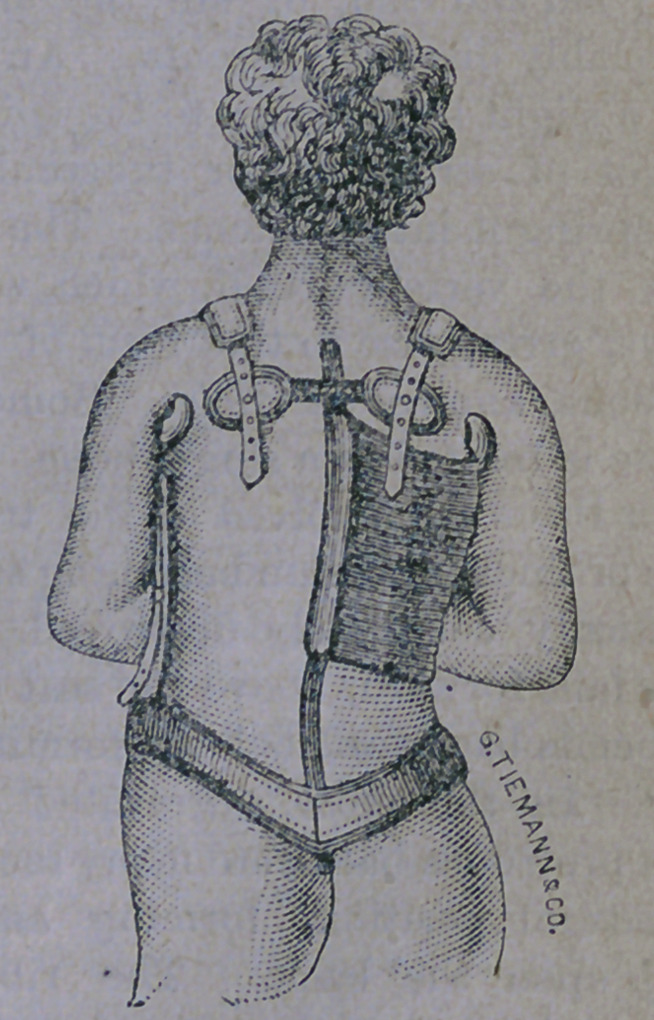


**Figure f3:**
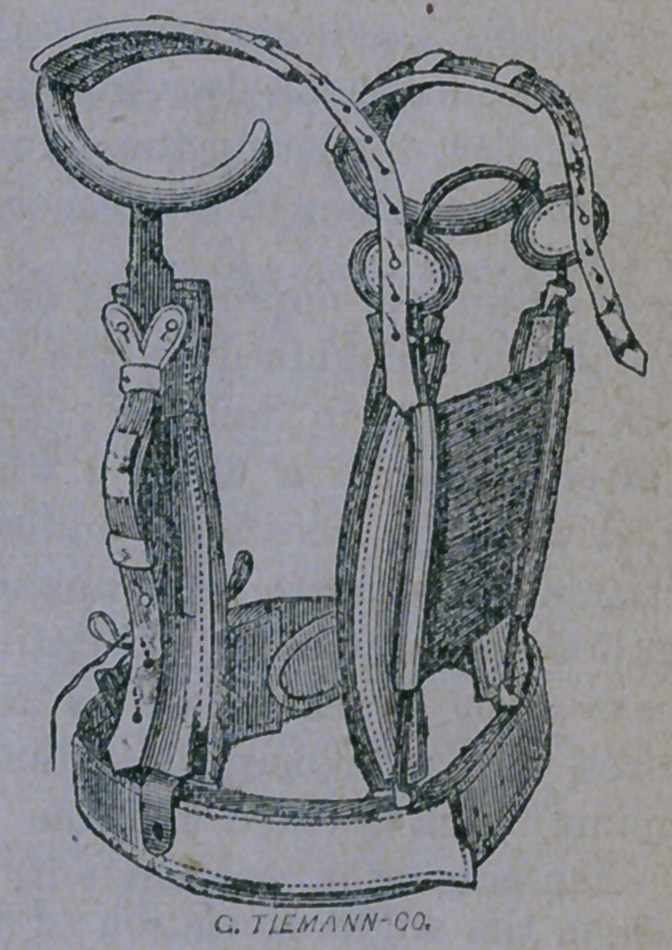


**Figure f4:**
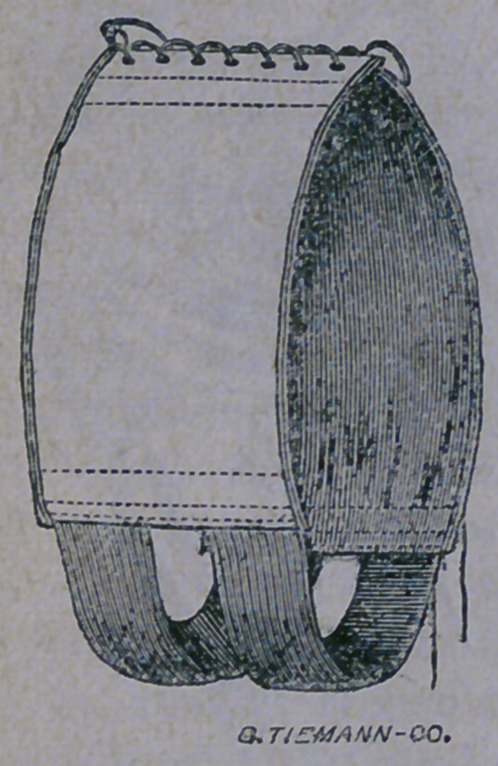


**Figure f5:**